# Evaluation of repeatability and agreement of two optical biometers for intraocular lens power calculation

**DOI:** 10.1038/s41598-024-73206-0

**Published:** 2024-09-27

**Authors:** Irene Martinez Alberquilla, Saga Svensson, Javier Ruiz-Alcocer, David Madrid-Costa, Alberto Dominguez-Vicent, Abinaya Priya Venkataraman

**Affiliations:** 1 Clinical and Experimental Eye Research Group, Faculty of Optics and Optometry, Universidad Complutense de Madrid, Madrid, Spain; 2 Department of Optometry and Vision, Faculty of Optics and Optometry, Universidad Complutense de Madrid, Madrid, Spain; 3 Unit of Optometry, Division of Eye and Vision, Department of Clinical Neuroscience, Karolinska Institute, Stockholm, 171 77 Sweden

**Keywords:** Repeatability, Agreement, IOL power calculation, Lenstar, Eyestar, Eye diseases, Lens diseases

## Abstract

The repeatability of two biometers (Lenstar-LS900 and Eyestar-900) to measure ocular parameters and intraocular lens (IOL) power calculation, and their agreement were evaluated. 134 eyes of 134 participants were measured thrice with each biometer. Axial length (AL), anterior chamber depth (ACD), lens thickness (LT) and keratometry (K) were evaluated. The IOL power was calculated using different formulas. The repeatability limit (RLimit), the mean differences (MD) and the limits of agreement (LoA) were calculated. The RLimits for all parameters were higher with Lenstar compared to Eyestar. RLimits were lower than 0.50 D except for Barrett Universal II (0.54 D) and Haigis (0.51 D) formulas with the Lenstar. Mean differences were lower than 0.01 mm for AL, ACD and LT, and lower than 0.03 D for K. MD ranged from 0 to 0.02 D for all formulas except for Barrett and Hill. When dividing the sample into subgroups (short, normal and long eyes), the MDs were similar for the IOL power and were lower than 0.03 D, except for the Barrett and Hill formulas. Both biometers provide repeatable biometry and IOL power calculations. The LoA interval for the IOL power calculation was between 0.75 and 1.50D, which was similar among the subgroups.

## Introduction

Intraocular lens (IOL) implantation is the gold-standard method to correct vision after cataract surgery or refractive lens exchange. It is known that small imbalances in IOL power calculation lead to residual refractive errors, inducing a marked deterioration in the retinal optical quality^1^. Residual refractive errors are one of the main causes of unsatisfactory visual rehabilitation after IOL implantation^2^. In a study from 2012, it was reported that in a cohort of over 17,000 eyes undergoing cataract surgery between 2008 and 2010, only 55% of the sample planned for emmetropia after cataract extraction reached that aim^3^. There has been an improving trend in the refractive outcome, and it has been reported that 73% of eyes achieving an absolute prediction error of 0.50 D or less and 94% of eyes achieving an absolute prediction error of 1.00 D or less in 2017^4^. The ultimate goal after cataract surgery is to achieve a given target refraction, whether it is emmetropia or any other refractive error^5^.

High precision in measuring ocular parameters and accurate power calculations are therefore key factors for visual success after IOL implantation. There are currently a wide variety of biometers available using different measurement principles. IOL power calculation is done with several formulas that require several inputs such as axial length (AL), keratometry or anterior chamber depth (ACD, distance between the corneal endothelium and anterior surface of the crystalline lens)^5,6^. However, the IOL power calculation is shown to be less accurate in short and long eyes compared to normal AL^7–9^.

Ocular biometers continue to evolve with advancements in technology, leading to improved accuracy, efficiency, and ease of use. Novel imaging techniques, such as optical coherence tomography (OCT) or optical low coherence reflectometry (OLCR), have further enhanced the capabilities of biometers, allowing for more comprehensive and detailed ocular measurements^10,11^. The Eyestar 900 biometer (Haag Streit AG, Koeniz, Switzerland) is based on swept-source OCT (SS-OCT) technology, and the Lenstar LS900 biometer (Haag Streit AG, Koeniz, Switzerland) is based on OLCR technology. Since their launch, there have been several studies that evaluated the performance of these biometers^12–17^. Several studies have also addressed the repeatability and agreement of these biometers separately in comparison to other devices^13,18–21^. The precision and agreement of these two biometers have also been compared for evaluating anterior segment parameters and AL^15,22^. However, to the best of the authors’ knowledge, there is no research evaluating these biometers to calculate the IOL power with different formulas.

Thus, the aim of this study was to evaluate the repeatability of two different biometers (Eyestar and Lenstar) to measure ocular biometric parameters and IOL power calculation, and to assess the agreement between devices. Additionally, these metrics were evaluated in short, normal, and long eyes.

## Methods

In this prospective study, subjects above 18 years of age who had no history of dry eye symptoms, ocular disorders, trauma, or surgeries were included. Signed informed consent was obtained from all participants. The study conformed to the principles of the Declaration of Helsinki and was approved by the Regional Ethics Committee (Swedish Ethical Review Authority). The demographic data collected from each participant included gender, age, and refraction.

### Instrumentation

Two different biometers with different optical technologies were analysed in this study: the Lenstar LS 900 biometer, software version V.2.5.2 (Haag Streit AG, Koeniz, Switzerland), is based on OLCR technology that uses an 820 nm super luminescent diode to measure biometric distances (ACD, LT and AL). Keratometric values are calculated using a light-emitting diode (LED) source of 950 nm to analyse the corneal curvature at 32 reference points distributed in two concentric circles of 2.30 and 1.65 mm diameters. The Eyestar 900 biometer, software version V2.2.0 (Haag Streit AG, Koeniz, Switzerland), is based on SS-OCT technology. It uses a wavelength of 1060 nm with a scan speed of 30 kHz for biometric values, and a LED source of 850 nm to obtain Dual Zone keratometry on measuring 32 points.

### Procedure

Biometers were calibrated before the measurements were taken. The measurements with both instruments were performed by the same examiner. One eye per participant was randomly selected for the study, and three measurements of all parameters were taken with each biometer. The biometric parameters evaluated are AL, ACD, lens thickness (LT) and keratometry (flat and steep meridians; K1 and K2).

The IOL power was calculated for each of the repeated measurements. Each biometer’s inbuild calculator (EyeSuite IOL) was used to calculate the IOL powers using the following formulas: Barrett Universal II, Haigis, Hill RBF 3.0, Hoffer Q, Holladay I, and SRK/T. In all formulas, the target refraction was set to emmetropia, and the chosen IOL model was the monofocal AcrySof IQ SN60WF, (Alcon Labs, Fort Worth, TX, USA).

### Statistical analysis

Descriptive analysis included mean and standard deviation (SD) of the evaluated parameters. Repeatability and agreement were calculated following the British and International Standards^23,24^. Repeatability was described using the repeatability limit (RLimit), calculated as 1.96√2 x S_w_ (where S_w_ is the within-subject standard deviation). RLimit represents the likely limits with which 95% of measurements should be within^23,24^. For all the ocular parameters, differences between each biometer were analysed with paired t test. The agreement was described using the mean difference between instruments and the limits of agreement (LoA), calculated as the mean difference ± 1.96 SD of the differences^23^. These calculations were performed for the whole sample, and also after dividing the participants into three subgroups based on their AL: short eyes (AL < 22.5 mm); normal eyes (AL between 22.5 and 24.5 mm), and long eyes (AL > 24.5 mm).

A sample size (n) calculation was performed taking into account both repeatability and agreement analysis. For the repeatability analysis, n was calculated considering the number of repeated measurements (m) and confidence level (CL)^24^ for the estimated Sw as $\:CL=\:\frac{1.96}{\sqrt{2\bullet\:n(m-1)}}$. For a CL of 0.10 and 3 repeated measurements, 96 eyes are required. For agreement, the following formula was used: $\:desired\:CI\:of\:LoA=1.96\bullet\:\sqrt{\frac{{3s}^{2}}{n}}$, where s is the SD of the differences. We considered the desired CI for the LoA in our study to be 0.1D for the IOL power (primary outcome). With this value and the s value obtained in a subset of the first 50 eyes, the minimum n value required was 99 eyes. Then, taking into account both n values, we considered that the minimum sample size should be at least 100 eyes.

## Results

134 eyes of 134 participants were included in the study: 67 right eyes and 67 left eyes. Mean age was 31.2 ± 12.5 years (Range: 20–66 years). 103 participants (76.9%) were women and 31 (23.1%) were men. The spherical equivalent for the entire sample was − 1.16 ± 2.89 D (-13.25 to + 6.37 D).

### Repeatability

Table 1 summarises the descriptive statistics and the repeatability metrics outcomes for the biometer parameters. The RLimits for all parameters evaluated in this study were higher with the Lenstar compared to the Eyestar. For AL, ACD and LT, the RLimits obtained with the Eyestar were lower than 0.07 mm, whereas the RLimits for the Lenstar ranged from 0.05 mm to 0.23 mm. RLimits for K1 and K2 were lower than 0.25 D for the Eyestar and lower than 0.50 D for the Lenstar.

**Table 1:** Descriptive statistics, repeatability metrics and agreement results for the anterior segment parameters.

Parameter	Instrument	Mean ± SD	RLimit	*P* value	Mean difference	LoA interval [range]
AL (mm)	Lenstar	23.80 ± 1.36	0.05	0.072	-0.01	0.14 [-0.07; 0.06]
	Eyestar	23.81 ± 1.35	0.02			
ACD (mm)	Lenstar	3.04 ± 0.36	0.21	0.503	0.01	0.51 [-0.25; 0.26]
	Eyestar	3.03 ± 0.35	0.05			
LT (mm)	Lenstar	3.81 ± 0.41	0.23	0.811	0.00	0.58 [-0.29; 0.29]
	Eyestar	3.81 ± 0.38	0.06			
K1 (D)	Lenstar	43.28 ± 1.36	0.31	0.084	0.02	0.64 [-0.30; 0.34]
	Eyestar	43.26 ± 1.37	0.21			
K2 (D)	Lenstar	44.35 ± 1.52	0.43	0.024	0.03	0.76 [-0.36; 0.41]
	Eyestar	44.32 ± 1.53	0.23			

ACD: anterior chamber depth; AL: axial length; K1: flat keratometry; K2: steep keratometry; LT: lens thickness; RLimit: repeatability limit; LOA: limit of agreement.

In all three subgroups analysed, the RLimits for AL were lower than 0.05 mm for both biometers (Table 2). For ACD and LT variables, Lenstar showed higher RLimits compared to Eyestar device for all subgroups.

**Table 2:** Descriptive statistics, repeatability metrics and agreement results for the anterior segment parameters divided into different axial length groups.

Parameter	Instrument		Mean ± SD	RLimit	Mean difference	LoA interval [range]
***Long eyes (n = 30)***						
AL (mm)	Lenstar		25.71 ± 1.08	0.03	-0.01	0.18 [-0.10; 0.08]
	Eyestar		25.71 ± 1.07	0.03		
ACD (mm)	Lenstar		3.26 ± 0.30	0.12	-0.02	0.20 [-0.12; 0.08]
	Eyestar		3.27 ± 0.30	0.04		
LT (mm)	Lenstar		3.80 ± 0.46	0.18	0.01	0.37 [-0.17; 0.20]
	Eyestar		3.79 ± 0.44	0.05		
***Normal eyes (n = 85)***						
AL (mm)	Lenstar		23.57 ± 0.55	0.05	-0.01	0.09 [-0.05; 0.04]
	Eyestar		23.58 ± 0.55	0.02		
ACD (mm)	Lenstar		3.01 ± 0.35	0.24	0.02	0.53 [-0.24; 0.29]
	Eyestar		2.99 ± 0.33	0.05		
LT (mm)	Lenstar		3.78 ± 0.39	0.24	-0.01	0.60 [-0.31; 0.29]
	Eyestar		3.79 ± 0.34	0.06		
***Short eyes (n = 19)***						
AL (mm)	Lenstar		21.91 ± 0.39	0.04	-0.01	0.19 [-0.11; 0.08]
	Eyestar		21.93 ± 0.38	0.03		
ACD (mm)	Lenstar		2.81 ± 0.31	0.18	-0.02	0.67 [-0.36; 0.32]
	Eyestar		2.83 ± 0.31	0.07		
LT (mm)	Lenstar		3.92 ± 0.42	0.27	-0.01	0.73 [-0.37; 0.35]
	Eyestar		3.92 ± 0.43	0.06		

ACD: anterior chamber depth; AL: axial length; LT: lens thickness; RLimit: repeatability limit; LOA: limit of agreement.

Table 3 summarises the descriptive statistics and repeatability metrics outcomes for the IOL power calculation for all formulas analysed. RLimits were lower than 0.50 D except for the Barrett Universal II and Haigis formulas calculated with the Lenstar. For the long eyes (Table 4), the RLimits for all formulas ranged between 0.48D to 0.65D for Lenstar and between 0.33D to 0.71D for the Eyestar. All RLimits measured in normal and short eyes were lower than 0.50 D, except for the Barrett Universal II in normal and Haigis for short eyes formulas calculated with the Lenstar.

**Table 3:** Descriptive statistics, repeatability metrics and agreement results for the IOL power calculated by different formulas.

Formula	Instrument	Mean ± SD	RLimit	Mean difference	LoA interval [range]
Barrett (D)	Lenstar	20.13 ± 3.86	0.54	0.16	1.26 [-0.47; 0.79]
	Eyestar	19.97 ± 3.83	0.40		
Haigis (D)	Lenstar	20.40 ± 3.99	0.51	0.00	0.99 [-0.50; 0.49]
	Eyestar	20.41 ± 3.98	0.42		
Hill (D)	Lenstar	20.45 ± 4.09	0.48	0.32	1.34 [-0.35; 0.99]
	Eyestar	20.13 ± 4.05	0.40		
Hoffer (D)	Lenstar	20.18 ± 4.15	0.47	0.00	1.02 [-0.51; 0.51]
	Eyestar	20.19 ± 4.14	0.41		
Holladay (D)	Lenstar	20.16 ± 3.98	0.50	-0.02	1.12 [-0.58; 0.54]
	Eyestar	20.18 ± 3.96	0.49		
SRK/T (D)	Lenstar	20.21 ± 3.90	0.48	0.00	0.99 [-0.50; 0.49]
	Eyestar	20.21 ± 1.35	0.37		

RLimit: limit of repeatability, LoA: limit of agreement.

**Table 4:** Descriptive statistics, repeatability metrics and agreement results for the IOL power calculated by different formulas and divided into different axial length groups.

				Repeatability	Agreement	
Formula	Instrument		Mean ± SD	RLimit	Mean difference	LoA interval [range]
***Long eyes***						
Barrett (D)	Lenstar		14.72 ± 2.79	0.65	0.14	1.25 [-0.49; 0.76]
	Eyestar		14.59 ± 2.76	0.47		
Haigis (D)	Lenstar		14.86 ± 2.91	0.58	-0.03	1.08 [-0.57; 0.51]
	Eyestar		14.89 ± 2.90	0.47		
Hill (D)	Lenstar		14.77 ± 3.25	0.55	0.28	1.44 [-0.44; 1.00]
	Eyestar		14.49 ± 3.11	0.53		
Hoffer (D)	Lenstar		14.43 ± 3.04	0.54	0.01	1.21 [-0.60; 0,61]
	Eyestar		14.43 ± 2.96	0.33		
Holladay (D)	Lenstar		14.60 ± 3.05	0.56	-0.03	1.45 [-0.76; 0.69]
	Eyestar		14.63 ± 2.97	0.71		
SRK/T (D)	Lenstar		14.68 ± 2.77	0.61	-0.03	1.34 [-0.70; 0.63]
	Eyestar		14.72 ± 1.07	0.42		
***Normal eyes***						
Barrett (D)	Lenstar		20.91 ± 1.80	0.53	0.14	1.23 [-0.48; 0.76]
	Eyestar		20.77 ± 1.79	0.40		
Haigis (D)	Lenstar		21.21 ± 1.90	0.48	0.00	0.91 [-0.46; 0.45]
	Eyestar		21.21 ± 1.90	0.40		
Hill (D)	Lenstar		21.35 ± 1.83	0.47	0.33	1.28 [-0.31; 0.97]
	Eyestar		21.01 ± 1.78	0.38		
Hoffer (D)	Lenstar		20.98 ± 1.98	0.48	-0.01	0.93 [-0.47; 0.45]
	Eyestar		20.99 ± 1.97	0.42		
Holladay (D)	Lenstar		20.95 ± 1.83	0.49	-0.02	0.98 [-0.51; 0.47]
	Eyestar		20.97 ± 1.81	0.40		
SRK/T (D)	Lenstar		20.91 ± 1.69	0.43	0.01	0.82 [-0.40; 0.42]
	Eyestar		20.90 ± 0.55	0.34		
***Short eyes***						
Barrett (D)	Lenstar		24.90 ± 2.84	0.41	0.28	1.30 [-0.37; 0.93]
	Eyestar		24.62 ± 2.84	0.32		
Haigis (D)	Lenstar		25.25 ± 3.03	0.52	0.03	1.18 [-0.57; 0.62]
	Eyestar		25.23 ± 3.07	0.41		
Hill (D)	Lenstar		25.42 ± 2.75	0.41	0.32	1.45 [-0.41; 1.04]
	Eyestar		25.11 ± 2.87	0.26		
Hoffer (D)	Lenstar		25.38 ± 3.01	0.26	0.02	1.14 [-0.55; 0.59]
	Eyestar		25.36 ± 2.98	0.45		
Holladay (D)	Lenstar		25.11 ± 2.78	0.45	0.02	1.18 [-0.57; 0.61]
	Eyestar		25.09 ± 2.91	0.41		
SRK/T (D)	Lenstar		25.25 ± 2.63	0.44	0.02	1.05 [-0.52; 0.52]
	Eyestar		25.25 ± 0.38	0.40		

RLimit: limit of repeatability; LoA: limit of agreement.

### Agreement

**Fig. 1:**
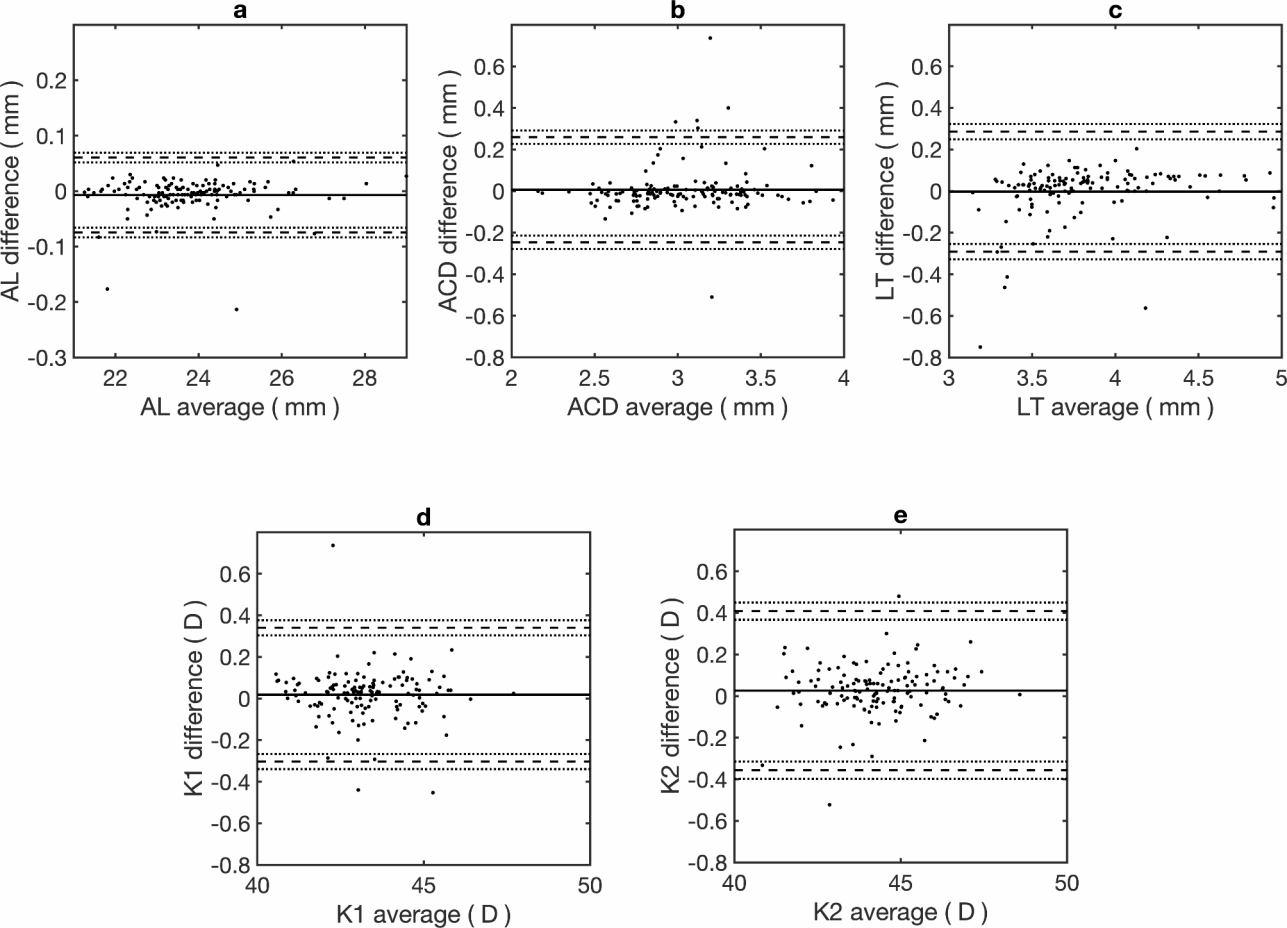
Agreement between the two biometers for the ocular parameter measurements. **a**: Axial length (AL), **b**: Anterior chamber depth (ACD), **c**: Lens thickness (LT), **d**: Flat keratometry (K1) and **e**: Steep keratometry (K2). Solid black dots: individual data points, Continuous black line: mean difference, dashed lines: lower and upper limits of agreement, dotted lines: lower and upper confidence intervals (95%) of each limits of agreement.

Table 1 summarises the mean difference and agreement results between instruments for the biometric parameters. Mean differences were lower than 0.01 mm for AL, ACD and LT, and lower than 0.03 D for K1 and K2. No significant differences were found between the two parameters for any of the parameters (*p* > 0.05). The shortest LoA interval was achieved for AL (0.14 mm), whereas ACD and LT showed LoA intervals of 0.51 and 0.58 mm, respectively. Keratometry LoA intervals were 0.64 and 0.76 D for K1 and K2, respectively. Bland Altman plot for agreement for all parameters are given in Fig. 1.

When dividing the sample into subgroups based on their axial length (Table 2), the mean differences ranged from 0.01 to 0.02 mm for AL, ACD and LT. For AL measurement with both biometers, the narrowest LoA interval was obtained for normal AL (0.09 mm), and it was similar for long and short eyes. For ACD and LT, long eyes had the narrowest LoA interval (0.20 and 0.37 mm).s.

Table 3 summarises the agreement results between instruments for the IOL power calculated by the different formulas for the overall sample. The mean differences for all formulas used ranged from 0 to 0.02 D, except for Barrett Universal II and Hill formulas. Regarding the LoA intervals, the Haigis and SRK/T formulas showed the narrowest interval (0.99D) and the Hill formula resulted in the widest interval (1.34D).

When dividing the sample into subgroups (Table 4), the mean difference for the IOL power was lower than 0.03 D, except for the Barrett Universal II and Hill formulas, where the differences were between 0.14 and 0.33 D. The LoA intervals were lower than 1.50 D for all formulas used, ranging from 0.72 to 1.45 D.

## Discussion

In the field of IOL power calculation, ensuring the accuracy and reliability of measurement techniques is paramount for achieving optimal visual outcomes following cataract surgery or refractive lens exchange procedures. Owing to this, the assessment of repeatability and agreement among different measurement methods has emerged as a crucial area of research interest. In the present study, we evaluated the repeatability of two different biometers (Eyestar and Lenstar) to measure ocular biometric parameters and IOL power calculation, and assessed the agreement between devices.

The accurate measurement of ocular parameters by biometers is essential for minimizing postoperative refractive errors^25^. Precise IOL power calculations based on reliable biometric data contribute to achieving the target refractive correction and reducing the need for additional corrective measures post-surgery. The results obtained showed good repeatability and agreement between devices.

Several studies have assessed the repeatability and agreement of these biometers to measure biometric parameters. Domínguez-Vicent et al.^15^ evaluated the repeatability and agreement of Eyestar 900 and Lenstar LS900 in several biometric parameters and found good repeatability. Shetty et al.^26^ also found good repeatability with the Lenstar LS 900 in measuring biometric parameters. Moreover, McAlinden et al.^27^ evaluated the repeatability of the Lenstar biometer in 102 eyes and found RLimits for AL, ACD, K1 and K2 of 0.05 mm, 0.06 mm, 0.29 D and 0.36 D, similar to our study, except for a lower value for ACD. Another study comparing two SS-OCT biometers and one OLCR also showed a higher repeatability performance with the SS-OCT biometers for AL, ACD, LT, K1 and K2 ^28^. In our study, Lenstar LS 900 obtained higher RLimits compared to Eyestar 900 for ACD and LT, K1 and K2 (Table 1), which is consistent with the literature. Venkataraman et al.^22^ performed repeatability analyses of anterior segment parameters, finding higher ACD RLimits for Lenstar (0.10) compared to Eyestar (0.05). When analysing the groups separately in the present study, long, normal and short eyes showed higher RLimits for ACD and LT measured with Lenstar compared to Eyestar (Table 2). These results seem to indicate that Lenstar biometer is less repeatable than Eyestar, especially in the measurements of ACD and LT, independent of the AL.

The agreement between these instruments had been previously studied for anterior segment parameter measurements. Domínguez-Vicent et al.^15^ found that the mean differences between devices for AL, ACD and LT were 0.004, 0.012 and − 0.024 mm, respectively, and our current corresponding results are − 0.01, 0.01 and 0.00 mm, which are similar. The narrowest LoA interval was obtained for AL, which also agrees with the results obtained in our study. In a similar study^22^, Eyestar and Lenstar showed a mean difference of 0.01 mm and a LoA interval of 0.25 mm for ACD parameter. When analysing the sample divided into different axial length groups, Lenstar LS 900 also obtained higher RLimits compared to Eyestar 900 for ACD and LT in all groups.

Regarding the repeatability of IOL power calculations (Table 3), all formulas with both devices obtained RLimits lower than or close to 0.50 D. Analysing the agreement for all the formulas, except for Barrett Universal II and Hill formulas, the mean difference was very small, almost close to zero. However, the LoA intervals were between 0.75 D and 1.25 D for Haigis, Hoffer, Holladay, and SRK/T formulas, and between 1.25 and 1.50 D for Barrett Universal II and Hill formulas Different IOL calculation formulas use different parameters to estimate postoperative ELP and calculate IOL power, with some formulas considering only AL and K values whereas others also consider ACD and LT values. This explains the difference in the agreement values for different formulas as ACD and LT values had larger agreement interval compared to AL.

To the best of our knowledge, there are no previous reports evaluating the agreement between these two devices for IOL power calculations. However, some studies evaluated Lenstar in comparison with other biometers. McAlinden et al.^27^ assessed the agreement between Lenstar and Aladdin biometer that are based on the principle of optical low coherence interferometry. For the IOL power calculation with SRK/T, Holladay 1, Hoffer Q and Haigis formulas the mean differences between devices were found to be between 0.01 and 0.02 D and the LoA intervals were within 0.50 D, concluding that both devices can be used interchangeably. These results seem to be comparable to our results for SRK/T, Holladay, Hoffer and Haigis formulas, with similar mean differences between devices. Additionally, other investigators^18^ also compared the consistency and accuracy in ocular biometric measurements and IOL power calculation using the Lenstar Ls 900 biometer and the IOLMaster, based on partial coherence interferometry technology. Mean differences between devices for Haigis, Hoffer Q, Holladay, and SRK/T formulas were between 0 and 0.04 D, similar to our results. Moreover, the agreement between Lenstar and Galilei G6 (combining a dual rotating Scheimpflug camera, a Placido disc topographer, and an optical coherence tomography-based optical A-scan) was assessed by Shin et al.^21^. The results showed poor agreement between IOL powers and for LT variable, suggesting that both devices cannot be interchangeable in clinical settings. Differences between studies could be due to different biometric technologies between devices.

The agreement between Eyestar and other biometers has also been studied^13,14,29^. Sorkin et al.^14^ compared the Eyestar 900 and IOLMaster 700 (both SS-OCT based biometers) to calculate the IOL power using the Barret Universal formula, and showed that the agreement was within ± 0.5 D in 98% of the eyes. Lender et al.^13^ compared the Eyestar and IOLMaster 700 and showed excellent agreement between the biometers. The IOL power calculations showed differences between 0.50 and 1.00D. Another study^29^ assessed the agreement between Eyestar 900 and Anterion, and showed that in 90.5% of the eyes, the IOL power calculations were within ± 0.5 D. Our study shows that the mean difference between Eyestar 900 and Lenstar for all IOL power formulas was lower than 0.50 D. The LoA intervals were higher for Barrett Universal II and Hill formulas with values around 1.25 D.

This study included only subjects with healthy eyes and no lens opacities. The previous studies discussed above include eyes undergoing cataract surgery. As our results are in line with these studies, we believe that similar results will also be obtained when comparing the Lenstar and Eyestar 900 in eyes with cataract.

## Conclusion

In conclusion, both biometers provide overall repeatable biometry and IOL power calculations in healthy eyes. The LoA interval for the different IOL power calculation formulas was between 0.75 and 1.50D, which was similar among the long, normal and short eyes.

## Data Availability

The datasets used and/or analysed during the current study are available from the corresponding author upon reasonable request.

## Abbreviations

IOL: Intraocular lens
AL: Axial length
ACD: Anterior chamber depth
OCT: Optical coherence tomography
OLCR: Optical low coherent reflectometry
SS-OCT: Swept-source optical coherence tomography
LT: Lens thickness
LED: Light-emitting diode
K1: Flat meridian keratometry
K2: Steep meridian keratometry
SD: Standard deviation
Sw: Within-subject standard deviation
RLimit: Repeatability limit
LoA: Limit of agreement
n: Sample size
m: Number of repeated measurements
CL: Confidence level
